# Websites and Resources for Cancer Family Caregivers

**Published:** 2013-07-01

**Authors:** Jo Hanson, Betty Ferrell, Marcia Grant

**Affiliations:** Ms. Hanson is a senior research specialist, Dr. Ferrell is a director and professor, and Dr. Grant is a professor, all at City of Hope National Medical Center in Duarte, California.

In the United States, over the past 12 months, more than 48 million people report serving as a family caregiver (FCG) for an adult loved one (National Alliance for Caregiving, 2009; Northouse, Katapodi, Schafenacker, & Weiss, 2012). The caregivers identify cancer as one of the most common illnesses of their care recipients. Remarkably, cancer FCGs provide an estimated 75% to 80% of their loved ones’ care over the disease trajectory. With predictions of 1.7 million new cancer diagnoses (Siegel, Naishadham, & Jemal, 2012; 2013) and more than 13 million cancer survivors (de Moor et al., 2013; O’Brien, Ness, Anderson, Sborov, & Foster, 2013), reliance on cancer FCGs will continue to increase.

Cancer health-care professionals have years of preparatory training for the physical, psychological, social, and spiritual complexities of cancer care. In contrast, FCGs, who provide the bulk of the care, are generally suddenly thrust into the caregiver role with minimal training and limited institutional support (van Ryn et al., 2011). Many FCGs feel anxious, ill prepared, and unsupported in their new roles (Ford, Catt, Chalmers, & Fallowfield, 2012) and look to the multidisciplinary oncology team—especially advanced practitioners (APs)—for information and support. To bridge the gap from being an ill-prepared, minimally supported caregiver to being a confident, knowledge-based caregiver, awareness and access to quality, reliable resources are essential. Increasingly, FCGs are going to the Internet to search for information. In fact, 8 out of 10 caregivers (79%) have access to the Internet. Of those, 88% look online for health information (Fox & Brenner, 2012).

The 2008 Institute of Medicine (IOM) report, Retooling for an Aging America: Building the Health Care Workforce, recommends patients and caregivers be considered essential parts of the health-care team and active participants in the health-care plan. Moreover, the report suggests, for caregivers to be effective members of the team, they need to have the necessary data, knowledge, and tools to provide high-quality care (IOM, 2008). To promote caregivers’ effective collaboration on the health-care team, resources need to include up-to-date, easily accessible information such as that featured on a page of the National Cancer Institute (NCI) website, entitled Family Caregivers in Cancer: Roles and Challenges (NCI, 2013).

## Caring for the Patient

To help FCGs get the support they need, APs must understand the complexity of problems and responsibilities associated with the cancer caregiver experience. Furthermore, APs must be prepared to assist FCGs in finding accurate, up-to-date information, especially online resources (see Tables 1 and 2).

Throughout the illness trajectory, caregivers deal with a multitude of new experiences: navigating a complex health-care system, procuring/dispensing medications, monitoring/managing symptoms, providing wound care, communicating with providers, providing emotional support, dealing with insurance, transporting to/from appointments, and managing side effects from treatment. The dynamic nature of cancer requires that the FCG always be "on call" to solve problems and make decisions as care needs change (Schubart, Kinzie, & Farace, 2008). Providing resources that consider these evolving needs promotes self-confidence in caregivers who are more prepared to anticipate and adjust to their shifting responsibilities. For example, the resources found on the American Cancer Society (ACS) website have extensive information on what to expect as a caregiver of a cancer patient (ACS, 2013).

## Caring for Self

Along with providing care for their loved one, FCGs have needs of their own: sleep, nutrition, exercise, social support, medical appointments/adherence to their own medical regimens, spiritual health, and respite from care responsibilities (Son et al., 2012). Too often these needs are pushed aside while caregivers focus on caring for the cancer patient. Studies have shown that FCGs frequently experience a decline in their own health and quality of life as a consequence of their caregiver role (Stenberg, Ruland, & Miaskowski, 2010). Encouragement from APs and access to reliable, high-quality caregiver-focused resources help FCGs take care of their own health needs and improve their quality of life. For instance, support groups such as those found through the CancerCare (CancerCare, 2013) and Cancer Support Community (Cancer Support Community, 2013) websites can be important resources to help strengthen the FCG’s psychological well-being.

## Conclusions

As the cancer care setting continues to shift from the hospital to the home, demands on FCGs accelerate. Increasingly, caregivers rely on health-care providers—especially APs—for guidance as they struggle to provide quality care for their loved one. Caregivers are eager for online resources that are accurate, pertinent, clear-cut, and easily accessible. Tables 1 and 2 give APs a list of resource websites to offer to FCGs as they care for their loved one throughout the cancer journey. Table 1 provides selected websites specifically developed for cancer FCGs. In addition, Table 2 offers valuable websites that will be helpful for all FCGs caring for a loved one experiencing health issues.

**Table 1:**
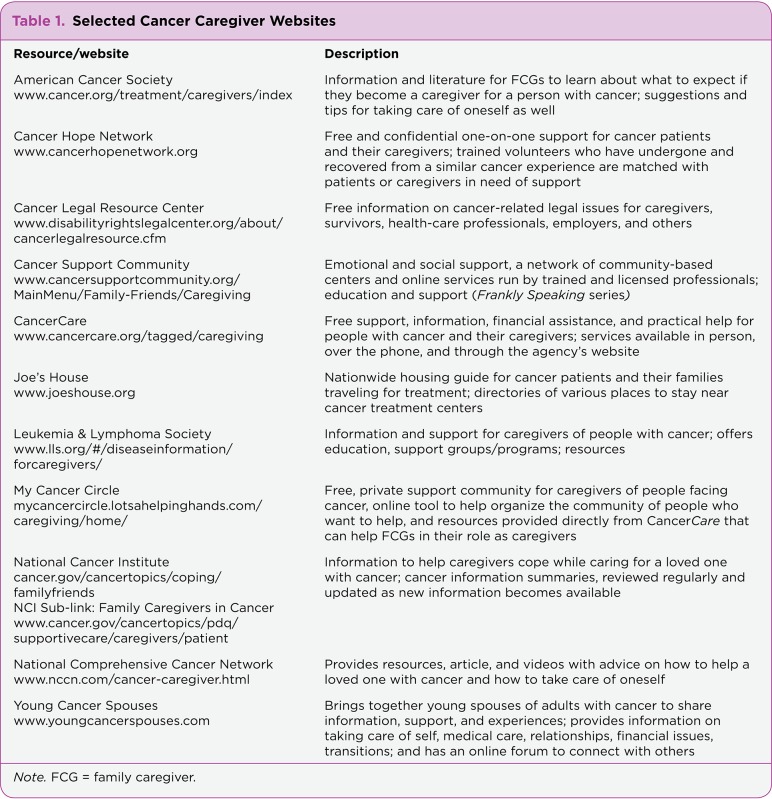
Table 1. Selected Cancer Caregiver Websites

**Table 2:**
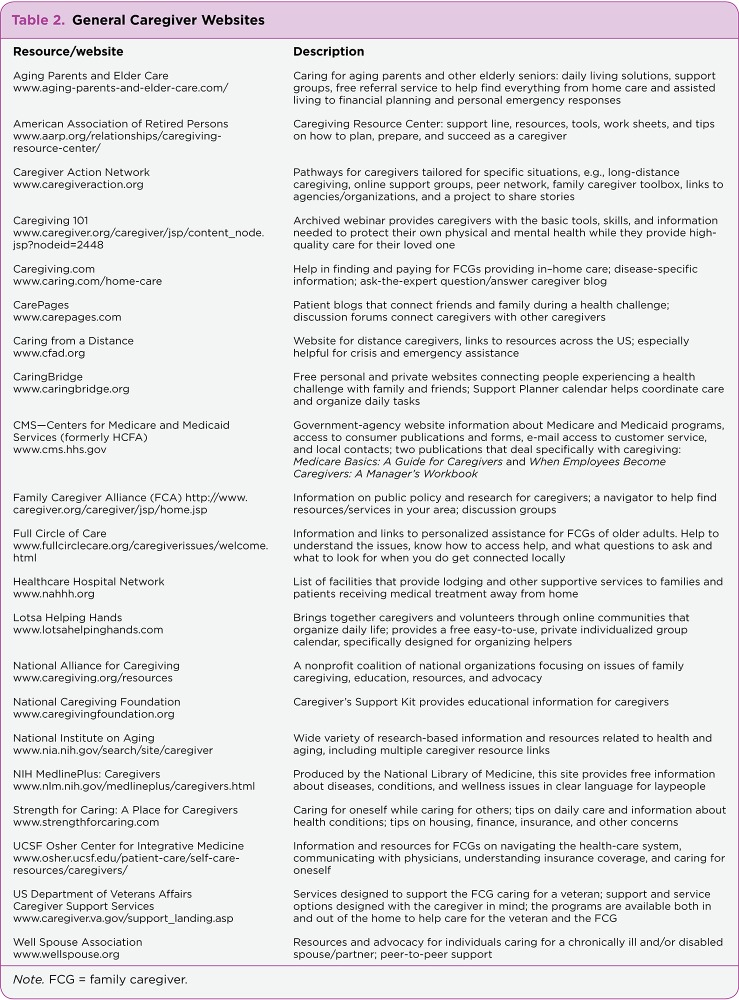
Table 2. General Caregiver Websites
